# Ventricular enlargement is associated with early Alzheimer’s disease pathophysiology

**DOI:** 10.1093/braincomms/fcag066

**Published:** 2026-03-07

**Authors:** Seyyed Ali Hosseini, Etienne Aumont, Nesrine Rahmouni, Marcel S Woo, Arthur C Macedo, Brandon Hall, Lydia Trudel, Tevy Chan, Jaime Fernandez Arias, Yi-Ting Wang, Stijn Servaes, Joseph Therriault, Yansheng Zheng, Kely Quispialaya Socualaya, Gleb Bezgin, Cécile Tissot, Delphine Oliva-Lopez, Robert Hopewell, Chris Hung-Hsin Hsiao, Catherine Saleh, Jenna Stevenson, Firoza Lussier, Liyong Wu, Min Chu, Sanjeev Chawla, Vladimir Fonov, Gassan Massarweh, Yasser Iturria-Medina, Jean-Paul Soucy, David A Rudko, Serge Gauthier, Thomas Karikari, Andréa Lessa Benedet, Nicholas J Ashton, Henrik Zetterberg, Maxime Montembeault, Paolo Vitali, Kaj Blennow, D Louis Collins, Jesse Klostranec, Tharick A Pascoal, Pedro Rosa-Neto

**Affiliations:** Translational Neuroimaging Laboratory, McConnell Brain Imaging Centre, Montreal Neurological Institute, McGill University, Montreal, Quebec H3A 2B4, Canada; Department of Neurology and Neurosurgery, McGill University, Montreal, Quebec H3A 1A1, Canada; The McGill University Research Center for Studies in Aging, Douglas Mental Health Institute, Montreal, Quebec H4H 1R3, Canada; Translational Neuroimaging Laboratory, McConnell Brain Imaging Centre, Montreal Neurological Institute, McGill University, Montreal, Quebec H3A 2B4, Canada; Department of Neurology and Neurosurgery, McGill University, Montreal, Quebec H3A 1A1, Canada; The McGill University Research Center for Studies in Aging, Douglas Mental Health Institute, Montreal, Quebec H4H 1R3, Canada; Translational Neuroimaging Laboratory, McConnell Brain Imaging Centre, Montreal Neurological Institute, McGill University, Montreal, Quebec H3A 2B4, Canada; Department of Neurology and Neurosurgery, McGill University, Montreal, Quebec H3A 1A1, Canada; The McGill University Research Center for Studies in Aging, Douglas Mental Health Institute, Montreal, Quebec H4H 1R3, Canada; Translational Neuroimaging Laboratory, McConnell Brain Imaging Centre, Montreal Neurological Institute, McGill University, Montreal, Quebec H3A 2B4, Canada; Department of Neurology and Neurosurgery, McGill University, Montreal, Quebec H3A 1A1, Canada; Translational Neurodegeneration Laboratory, Department of Neurology, University Medical Center Hamburg-Eppendorf, Hamburg 20246, Germany; Institute of Neuroimmunology and Multiple Sclerosis, University Medical Center Hamburg-Eppendorf, Hamburg 20246, Germany; Translational Neuroimaging Laboratory, McConnell Brain Imaging Centre, Montreal Neurological Institute, McGill University, Montreal, Quebec H3A 2B4, Canada; Department of Neurology and Neurosurgery, McGill University, Montreal, Quebec H3A 1A1, Canada; The McGill University Research Center for Studies in Aging, Douglas Mental Health Institute, Montreal, Quebec H4H 1R3, Canada; Translational Neuroimaging Laboratory, McConnell Brain Imaging Centre, Montreal Neurological Institute, McGill University, Montreal, Quebec H3A 2B4, Canada; Department of Neurology and Neurosurgery, McGill University, Montreal, Quebec H3A 1A1, Canada; The McGill University Research Center for Studies in Aging, Douglas Mental Health Institute, Montreal, Quebec H4H 1R3, Canada; Translational Neuroimaging Laboratory, McConnell Brain Imaging Centre, Montreal Neurological Institute, McGill University, Montreal, Quebec H3A 2B4, Canada; Department of Neurology and Neurosurgery, McGill University, Montreal, Quebec H3A 1A1, Canada; The McGill University Research Center for Studies in Aging, Douglas Mental Health Institute, Montreal, Quebec H4H 1R3, Canada; Translational Neuroimaging Laboratory, McConnell Brain Imaging Centre, Montreal Neurological Institute, McGill University, Montreal, Quebec H3A 2B4, Canada; Department of Neurology and Neurosurgery, McGill University, Montreal, Quebec H3A 1A1, Canada; The McGill University Research Center for Studies in Aging, Douglas Mental Health Institute, Montreal, Quebec H4H 1R3, Canada; Translational Neuroimaging Laboratory, McConnell Brain Imaging Centre, Montreal Neurological Institute, McGill University, Montreal, Quebec H3A 2B4, Canada; Department of Neurology and Neurosurgery, McGill University, Montreal, Quebec H3A 1A1, Canada; The McGill University Research Center for Studies in Aging, Douglas Mental Health Institute, Montreal, Quebec H4H 1R3, Canada; Translational Neuroimaging Laboratory, McConnell Brain Imaging Centre, Montreal Neurological Institute, McGill University, Montreal, Quebec H3A 2B4, Canada; Department of Neurology and Neurosurgery, McGill University, Montreal, Quebec H3A 1A1, Canada; The McGill University Research Center for Studies in Aging, Douglas Mental Health Institute, Montreal, Quebec H4H 1R3, Canada; Translational Neuroimaging Laboratory, McConnell Brain Imaging Centre, Montreal Neurological Institute, McGill University, Montreal, Quebec H3A 2B4, Canada; Department of Neurology and Neurosurgery, McGill University, Montreal, Quebec H3A 1A1, Canada; The McGill University Research Center for Studies in Aging, Douglas Mental Health Institute, Montreal, Quebec H4H 1R3, Canada; Translational Neuroimaging Laboratory, McConnell Brain Imaging Centre, Montreal Neurological Institute, McGill University, Montreal, Quebec H3A 2B4, Canada; Department of Neurology and Neurosurgery, McGill University, Montreal, Quebec H3A 1A1, Canada; The McGill University Research Center for Studies in Aging, Douglas Mental Health Institute, Montreal, Quebec H4H 1R3, Canada; Translational Neuroimaging Laboratory, McConnell Brain Imaging Centre, Montreal Neurological Institute, McGill University, Montreal, Quebec H3A 2B4, Canada; Department of Neurology and Neurosurgery, McGill University, Montreal, Quebec H3A 1A1, Canada; The McGill University Research Center for Studies in Aging, Douglas Mental Health Institute, Montreal, Quebec H4H 1R3, Canada; Translational Neuroimaging Laboratory, McConnell Brain Imaging Centre, Montreal Neurological Institute, McGill University, Montreal, Quebec H3A 2B4, Canada; Department of Neurology and Neurosurgery, McGill University, Montreal, Quebec H3A 1A1, Canada; The McGill University Research Center for Studies in Aging, Douglas Mental Health Institute, Montreal, Quebec H4H 1R3, Canada; Translational Neuroimaging Laboratory, McConnell Brain Imaging Centre, Montreal Neurological Institute, McGill University, Montreal, Quebec H3A 2B4, Canada; Department of Neurology and Neurosurgery, McGill University, Montreal, Quebec H3A 1A1, Canada; The McGill University Research Center for Studies in Aging, Douglas Mental Health Institute, Montreal, Quebec H4H 1R3, Canada; Department of Molecular Biophysics and Integrated Bioimaging, Lawrence Berkeley National Laboratory, Berkeley, CA 94720, USA; Translational Neuroimaging Laboratory, McConnell Brain Imaging Centre, Montreal Neurological Institute, McGill University, Montreal, Quebec H3A 2B4, Canada; Department of Neurology and Neurosurgery, McGill University, Montreal, Quebec H3A 1A1, Canada; The McGill University Research Center for Studies in Aging, Douglas Mental Health Institute, Montreal, Quebec H4H 1R3, Canada; Translational Neuroimaging Laboratory, McConnell Brain Imaging Centre, Montreal Neurological Institute, McGill University, Montreal, Quebec H3A 2B4, Canada; The McGill University Research Center for Studies in Aging, Douglas Mental Health Institute, Montreal, Quebec H4H 1R3, Canada; Department of Neurology and Neurosurgery, McGill University, Montreal, Quebec H3A 1A1, Canada; Department of Neurology and Neurosurgery, McGill University, Montreal, Quebec H3A 1A1, Canada; The McGill University Research Center for Studies in Aging, Douglas Mental Health Institute, Montreal, Quebec H4H 1R3, Canada; Department of Psychiatry, School of Medicine, University of Pittsburgh, Pittsburgh, PA 15213, USA; Translational Neuroimaging Laboratory, McConnell Brain Imaging Centre, Montreal Neurological Institute, McGill University, Montreal, Quebec H3A 2B4, Canada; Translational Neuroimaging Laboratory, McConnell Brain Imaging Centre, Montreal Neurological Institute, McGill University, Montreal, Quebec H3A 2B4, Canada; Department of Radiology, Perelman School of Medicine at the University of Pennsylvania, Philadelphia, PA 19104, USA; Department of Neurology and Neurosurgery, McGill University, Montreal, Quebec H3A 1A1, Canada; Department of Neurology and Neurosurgery, McGill University, Montreal, Quebec H3A 1A1, Canada; Department of Neurology and Neurosurgery, McGill University, Montreal, Quebec H3A 1A1, Canada; Department of Neurology and Neurosurgery, McGill University, Montreal, Quebec H3A 1A1, Canada; Department of Neurology and Neurosurgery, McGill University, Montreal, Quebec H3A 1A1, Canada; Department of Biomedical Engineering, McGill University, Montreal, Quebec H3A 2B4, Canada; Department of Neurology and Neurosurgery, McGill University, Montreal, Quebec H3A 1A1, Canada; The McGill University Research Center for Studies in Aging, Douglas Mental Health Institute, Montreal, Quebec H4H 1R3, Canada; Department of Psychiatry, School of Medicine, University of Pittsburgh, Pittsburgh, PA 15213, USA; Department of Psychiatry and Neurochemistry, Institute of Neuroscience and Physiology, the Sahlgrenska Academy, University of Gothenburg, Mölndal 413 45, Sweden; Department of Neurology, School of Medicine, University of Pittsburgh, Pittsburgh, PA 15213, USA; Department of Psychiatry and Neurochemistry, Institute of Neuroscience and Physiology, the Sahlgrenska Academy, University of Gothenburg, Mölndal 413 45, Sweden; Department of Psychiatry and Neurochemistry, Institute of Neuroscience and Physiology, the Sahlgrenska Academy, University of Gothenburg, Mölndal 413 45, Sweden; Wallenberg Centre for Molecular Medicine, University of Gothenburg, Gothenburg 413 45, Sweden; King’s College London, Institute of Psychiatry, Department of Psychology and Neuroscience, Maurice Wohl Institute Clinical Neuroscience Institute, London SE5 9NU, UK; NIHR Biomedical Research Centre for Mental Health and Biomedical Research Unit for Dementia at South London and Maudsley NHS Foundation, London, UK; Department of Psychiatry and Neurochemistry, Institute of Neuroscience and Physiology, the Sahlgrenska Academy, University of Gothenburg, Mölndal 413 45, Sweden; Clinical Neurochemistry Laboratory, Sahlgrenska University Hospital, Mölndal SE-431 80, Sweden; Department of Neurodegenerative Disease, UCL Institute of Neurology, London, UK; UK Dementia Research Institute at UCL, London, UK; Hong Kong Center for Neurodegenerative Diseases, Division of Life Science, Hong Kong 999077, China; Wisconsin Alzheimer’s Disease Research Center, University of Wisconsin School of Medicine and Public Health, University of Wisconsin-Madison, Madison, WI 53792, USA; The McGill University Research Center for Studies in Aging, Douglas Mental Health Institute, Montreal, Quebec H4H 1R3, Canada; Department of Neurology and Neurosurgery, McGill University, Montreal, Quebec H3A 1A1, Canada; Department of Psychiatry and Neurochemistry, Institute of Neuroscience and Physiology, the Sahlgrenska Academy, University of Gothenburg, Mölndal 413 45, Sweden; Clinical Neurochemistry Laboratory, Sahlgrenska University Hospital, Mölndal SE-431 80, Sweden; Paris Brain Institute, ICM, Pitié-Salpêtrière Hospital, Sorbonne University, Paris 75013, France; Division of Life Sciences and Medicine, Department of Neurology, Neurodegenerative Disorder Research Center, Institute on Aging and Brain Disorders, University of Science and Technology of China and First Affiliated Hospital of USTC, Hefei 230001, P.R. China; Department of Neurology and Neurosurgery, McGill University, Montreal, Quebec H3A 1A1, Canada; Department of Diagnostic and Interventional Neuroradiology, Montreal Neurological Institute and Hospital, McGill University, Montreal, Quebec H3A 2B4, Canada; Department of Psychiatry, School of Medicine, University of Pittsburgh, Pittsburgh, PA 15213, USA; Translational Neuroimaging Laboratory, McConnell Brain Imaging Centre, Montreal Neurological Institute, McGill University, Montreal, Quebec H3A 2B4, Canada; Department of Neurology and Neurosurgery, McGill University, Montreal, Quebec H3A 1A1, Canada; The McGill University Research Center for Studies in Aging, Douglas Mental Health Institute, Montreal, Quebec H4H 1R3, Canada; The Peter O’Donnell Jr. Brain Institute (OBI), University of Texas Southwestern Medical Centre (UTSW), Dallas, TX 75390, USA

**Keywords:** ventricular enlargement, CSF clearance, choroid plexus, protein aggregation, Alzheimer’s disease

## Abstract

Alzheimer’s disease (AD) is characterized by progressive brain changes, including protein aggregation and structural changes. Cerebrospinal fluid (CSF) system abnormalities, such as ventricular dilation, increased choroid plexus volume or positron emission tomography (PET) ligand uptake in the CSF, have also been consistently described. We aimed to examine whether changes in CSF production and clearance might be associated with brain protein aggregation across biological stages of Alzheimer’s disease. We hypothesized an association between brain protein aggregation and changes on the CSF system. We examined 378 individuals from the Translational Biomarkers in Aging and Dementia (TRIAD) cohort with T1-weighted magnetic resonance imaging (MRI), amyloid-PET and tau-PET assessments. We assessed the lateral ventricle and choroid plexus volumes, both corrected for intracranial volume, in the MRI native space. Non-specific ventricular tracer standardized uptake value ratio (SUVR), derived from amyloid- and tau-PET images, was used as an indirect marker of choroid plexus-related clearance activity and served as a metric of CSF dynamics. Linear models tested associations amongst lateral ventricular volume (reflecting CSF space enlargement), choroid plexus volume (reflecting secretory tissue morphology) and ventricular SUVR (reflecting tracer activity within the CSF compartment and serving as an indirect marker of choroid plexus-related clearance function and CSF dynamics) with Aβ and tau aggregations. Analyses were restricted to within-modality associations, relating ventricular radioactivity to cortical pathology for each PET tracer. We found that when considered independently, larger ventricular and choroid plexus volumes were associated with higher neocortical Aβ-PET SUVR, particularly in the precuneus and cingulate cortices. Additionally, lower ventricular radioactivity (derived from amyloid-PET) showed strong negative associations in the dorsal apex of the neocortex. However, when all three ventricular parameters were included in the same model, these effects were mediated by ventricular volume. By contrast, the effect of the ventricular parameters on tau load was mediated by Aβ in the neocortex. Therefore, ventricular enlargement appears to be associated with Aβ load. Distinct from neurodegeneration, changes in ventricular parameters, particularly ventricular volume, are associated with upstream Alzheimer’s disease pathophysiology. While ventricular volume significantly mediated ventricular amyloid clearance, no such effect was observed for tau, suggesting distinct clearance mechanisms for these pathologies in Alzheimer’s disease.

## Teaser

Hosseini *et al*. report that cerebrospinal fluid (CSF) clearance measures, including ventricular and choroid plexus changes, are linked to early amyloid-beta accumulation in Alzheimer’s disease, which in turn drives tau pathology. Their findings suggest CSF clearance dysfunction is an upstream contributor to disease progression, not merely a downstream neurodegenerative marker.

## Graphical Abstract

**Graphical Abstract:**
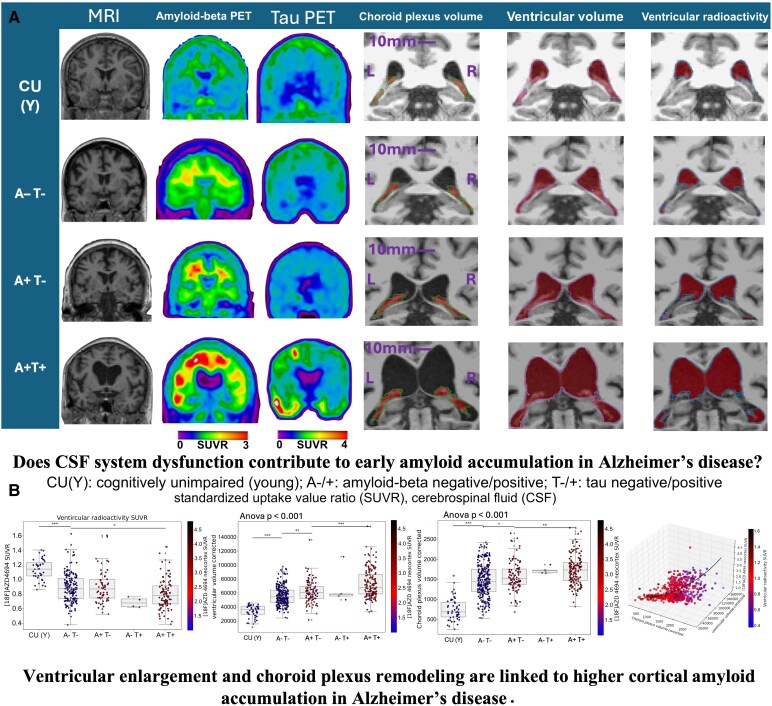

## Introduction

The cerebrospinal fluid (CSF) system refers to all components involved in CSF production, circulation and absorption,^1^ comprising the choroid plexus, which produces CSF within the ventricles^2^; the ventricular network that distributes CSF throughout the brain^3^; the arachnoid granulations responsible for reabsorption into the venous circulation^4^; and the glymphatic and perivascular pathways that facilitate the exchange and clearance of interstitial solutes.^5^ Abnormalities in the CSF clearance system and related structures are conceptualized as neurodegenerative in their association with Alzheimer’s disease pathophysiology.^6^ It has also been proposed that dysfunction of the CSF clearance system might facilitate early disease processes by disrupting extracellular fluid dynamics and reducing clearance of brain metabolic waste.^7-9^

In fact, neuropathology studies have revealed that the choroid plexus’ complex network of capillaries and secretory cells undergo epithelial atrophy, stromal fibrosis, vessel thickening and inflammatory changes.^10^ In addition, choroid plexus enlargement has also been associated with cognitive decline and increased amyloid-beta (Aβ) cortical load.^11^ Recently, *in vivo* assessment of CSF dynamics using the magnitude of positron emission tomography (PET) signal in the lateral ventricles [ventricular radioactivity (VR)] has shown associations between reduced choroid plexus function and increased cortical Aβ formation.^12^ Independent replication of these findings supports the notion that CSF production abnormalities are linked to Aβ aggregation.^13,14^ Although this VR may be influenced by several factors, including choroid plexus activity, ependymal permeability and tracer diffusion, it provides a useful indirect measure of CSF-related clearance function.^12-14^

Ventricular enlargement is another CSF clearance system abnormality frequently described in neuroimaging studies of ageing and Alzheimer’s disease. It has often been associated with brain atrophy and cognitive decline.^15,16^ Ependymal gliosis, periventricular oedema and declines in white matter tract integrity are pathological features associated with ventricular enlargement.^17^ Conceptualized as a downstream structural abnormality, ventricular enlargement represents a reliable clinical trial outcome measure with stronger effect size and sensitivity than hippocampal or total brain volume reduction for diagnosing Alzheimer’s disease.^18^ Although ventricular enlargement has been described as related to Alzheimer’s disease pathophysiology, its impact on early Alzheimer’s disease pathophysiology has not been thoroughly addressed.^19^

A growing body of evidence highlights the potential interplay between CSF clearance system abnormalities and early Alzheimer’s disease pathophysiology.^20-23^ More precisely, it is thought that enlargement of the ventricles may causes the choroid plexus to produce more CSF to fill its cavity. However, in Alzheimer’s disease, the choroid plexus function might also be compromised.^13^ Therefore, due to their coexistence in the same individual, CSF-related abnormalities can potentially interact amongst themselves, ultimately resulting in accelerated amyloid plaque or tau tangle formation due to compromised CSF clearance.^24^ To test this hypothesis, one should apply multiparametric approaches to examine the direct and indirect effects of the changes in the ventricular parameters on the aggregation of proteins in the brain. To do so, we assessed structural magnetic resonance imaging (MRI) for ventricular and choroid plexus enlargement and PET tracers for Αβ deposition and tau load. We estimated the lateral ventricle and choroid plexus volumes (CPVs). Additionally, the lateral ventricular PET signal was quantified using both amyloid ([^18^F]AZD4694) and tau ([^18^F]MK6240) tracers. Because these tracers are diluted in CSF, their signal in the ventricular space depends on blood-to-CSF transport and ventricular clearance, providing an indirect yet reproducible index of CSF dynamics.^12^ Specifically, we tested the hypothesis that ventricular enlargement facilitates Aβ accumulation.

## Materials and methods

### Participants

In this study, we initially included 472 individuals from the Translational Biomarkers in Aging and Dementia (TRIAD) cohort at McGill University^25^ (52 young individuals under 25 years old {cognitively unimpaired young [CU(Y)]}, 267 cognitively unimpaired (CU) older adults, 59 individuals with mild cognitive impairment (MCI), 65 individuals with typical Alzheimer’s disease dementia and 29 individuals with atypical Alzheimer’s disease dementia). Out of 472 individuals, 403 individuals had MRI, 384 individuals had [^18^F]MK6240 PET imaging and 392 had [^18^F]AZD4694 PET imaging. We ensured that all participants had T1 MRI and both an amyloid- and tau-PET scan for all analysis. Additionally, we systematically excluded participants with abnormal ventricular enlargement unrelated to Alzheimer’s disease, defined by an Evans’ Index (EI) > 0.3.^26^ A detailed description of how EI was calculated from the FreeSurfer segmentation using a semi-automated Python workflow, which measured the maximum frontal horn width within the anterior third of the lateral ventricles and divided it by the internal skull diameter estimated from the head-included T1-weighted MRI, is provided in the Supplementary material, section ‘Evans’ Index measurement’, along with representative examples of subjects with EI > 0.3 and EI < 0.3 shown in Supplementary Figs. 1 and 2. According to these criteria, the final sample size was 378 participants comprising: 52 CU(Y), 166 amyloid-negative/tau-negative (A−T−), 64 amyloid-positive/tau-negative (A+T−), 4 A−T+ and 99 amyloid-positive/tau-positive (A+T+).

Participants in the CU group exhibited no objective cognitive impairment. CU participants had a Clinical Dementia Rating (CDR) score of 0, whereas those with MCI had a CDR score of 0.5 and demonstrated objective cognitive impairment with relatively preserved performance in activities of daily living.^27^ Participants with Alzheimer’s disease dementia had a CDR score of 1 or 2 and met the standard diagnostic criteria for probable Alzheimer’s disease.^28^ A multidisciplinary team of neurologists, neuropsychologists and nurses classified individuals as MCI individuals and Alzheimer’s disease dementia patients based on the National Institute on Aging–Alzheimer’s Association (NIA-AA) criteria for MCI due to Alzheimer’s disease^29^ and for Alzheimer’s disease dementia,^30^ respectively. The study obtained approval from the Montreal Neurological Institute PET working committee and the Douglas Mental Health University Institute Research Ethics Board (IUSMD 16-60). All participants provided informed consent after full disclosure of research procedures.

### Imaging acquisition and processing

A 3 Tesla Siemens MAGNETOM MRI scanner with a standard head coil was utilized to acquire a T1-weighted image using an Ultrafast Gradient Echo 3D sequence (isotropic 1 mm voxels, repetition time, 2300 ms; echo time, 2.96 ms; field of view, 256 mm; flip angle, 9°). A brain-dedicated Siemens high-resolution research tomograph was used for [^18^F]AZD4694, and [^18^F]MK6240 PET scan acquisitions. For the amyloid-PET scan, the mean injected radioactive dose was 6.45 ± 0.61 mCi, whereas the tau-PET scan used a mean injected dose of 6.28 ± 0.74 mCi. Tau-PET images were captured 90–110 min post [^18^F]MK6240 intravenous bolus injection with four frames (4 × 300 s), as previously reported.^31^ Aβ-PET images were acquired 40–70 min after the [^18^F]AZD4694 intravenous injection with three frames (3 × 600 s). For both PET tracer images, the reconstruction was performed using a sequential subset expectation–maximization algorithm on a 4D volume. A 6-min transmission scan using a rotating 137Cs point source was conducted after each PET acquisition to correct for motion, dead time, decay and both random and scattered coincidences.

### Processing and tissue standardized uptake value ratio derivation

PET images were then linearly registered to T1-weighted MRI image space and subsequently linearly and nonlinearly registered to the Alzheimer’s Disease Neuroimaging Initiative (ADNI) standard template using ANTs (version 2.2.0). Additionally, PET images were spatially smoothed to achieve a full width at half maximum (FWHM) of 8 mm for the modulation transfer function curve. After transferring the images to ADNI space, a mask of stripped meninges, created using FSL (version 6.0.2), was applied to the PET images, followed by blurring to prevent meningeal spill-over into adjacent brain areas and ensure accurate signal localization.^32^ The standardized uptake value ratio (SUVR) in [^18^F]MK6240 PET was determined using the inferior cerebellum grey matter as the reference region.^33^ For [^18^F]AZD4694 SUVR, the full cerebellum grey matter served as the reference region.^34^ The neocortex was used for the global [^18^F]AZD4694 SUVR composite, including the precuneus, prefrontal, orbitofrontal, parietal, temporal and cingulate cortices.^34^ The threshold for [^18^F]AZD4694 SUVR positivity was set at an SUVR > 1.55, as previously described.^34^ The temporal meta-region of interest (ROI) was used for the global [^18^F]MK6240 SUVR composite, including the entorhinal cortex, the amygdala, the hippocampus and the parahippocampal, fusiform, lingual, inferior temporal and middle temporal gyri.^35^ The threshold for [^18^F]MK6240 SUVR positivity was established at an SUVR > 1.18.^36^

### Volumetric assessments

We obtained lateral ventricles and choroid plexus regions in native space for each MRI using FreeSurfer (version 7.4.1).^37^ This fully automated segmentation method is specifically tailored for enhanced detection and segmentation of grey matter, white matter and other brain’s structures. The segmentation of the lateral ventricles and the choroid plexus within the lateral ventricles was used to quantify ventricular and CPVs.

### Derivation of ventricular radioactivity

The lateral ventricles served as ROIs to quantify VR from each of the PET images. To control for the specificity of tracer binding to tau, amyloid and other potential targets in the choroid plexus, we excluded the choroid plexus from the ventricles before the calculations. After removal of the choroid plexus, the ventricular masks underwent ∼2 mm morphological erosion to reduce immediate boundary-related partial-volume interactions while preserving sufficient ventricular volume (VV). Additionally, the PET images were reconstructed using a four-dimensional ordered subset expectation maximization (OSEM) algorithm, which reduces spill-over artefacts prior to SUVR estimation.^38^ The ventricles, filled with CSF, are considered as non-specific binding areas in PET imaging, where radiotracer accumulation is subject to bulk diffusion without tracer-specific molecular targets. Modulation of the levels of radioactivity in the ventricles may be affected by the rate of CSF production due to choroid plexus function.^12^

### Fluid biomarkers

A subset of participants underwent CSF and plasma sampling, as previously described.^34,39^ CSF was collected by lumbar puncture and analysed for Aβ40 and Aβ42 concentrations using the Lumipulse^®^ G platform (Fujirebio). Plasma Aβ40 and Aβ42 were measured using the Simoa^®^ HD-X platform (Quanterix). These assays provided quantitative measures of Aβ species in both biofluids to support the interpretation of PET imaging and cognitive findings.

### Statistical analysis

Statistical analyses were conducted using in-house Python code. For group comparisons across the amyloid and tau positivity (A/T) groups, one-way analysis of variance (ANOVA) with the SciPy library and followed by *post hoc* comparisons using Tukey’s honestly significant difference (HSD) test were implemented, and results were corrected for multiple comparisons, using the false discovery rate (FDR) controlled by the Benjamini–Hochberg procedure (*q* < 0.05).

Linear regression lines with associated *P*-values, Spearman correlation coefficients (*ρ*) and coefficients of determination (*R*^2^) were added to the scatter plots using the Matplotlib and SciPy libraries. Results were corrected for multiple comparisons using the Benjamini–Hochberg procedure to control the FDR. Regression analyses were conducted with and without CU(Y) participants to account for potential age-related effects.

Voxel-based linear regression analysis explored the relationship between different ventricular parameters and amyloid- and tau-PET load using the MATLAB package VoxelStats.^40^ All models included age, sex and APOE4 carriership as covariates. Analyses were adjusted for multiple comparisons using the random field theory method^41^ with a significance threshold set at *P* < 0.001. The resulting t-map from VoxelStats was overlaid on a structural template surface using the BrainNet Viewer package (version 1.63)^42^ in MATLAB (version 2018a).

The mediation pathway analysis explored relationships between ventricular parameters and Aβ and tau loads through linear regression, calculating standardized beta coefficients and *P*-values. Covariates [age, sex, APOE, intracranial volume (ICV)] were encoded, standardized and included in the analysis, with results visualized using NetworkX and Matplotlib libraries.

The *Z*-scores of ventricular parameters across A/T stages were calculated using the mean and standard deviation of CU(Y) participants as the reference group with the NumPy library.

The CPV and VV were corrected for ICV using linear regression, with the residuals computed using the statsmodels library, and subsequently used in all analyses. Correcting choroid plexus and VVs for ICV accounts for head size variability, ensuring changes reflect pathology rather than anatomical differences.^43^ To further control for brain atrophy-related confounding effects and differentiate between ventricular enlargement due to atrophy-related changes and increased protein aggregation,^44^ we corrected VV by grey matter volume to isolate its relationship with protein deposition in voxel-wise analysis when including all ventricular parameters in the same model.

## Results

In the current study, we systematically excluded participants with abnormal ventricular enlargement who belonged to the A−T− CU group (*n* = 12, ≈2%). The final sample size included in the current study was 378 participants, comprising 52 CU(Y), 166 A−T−, 64 A+T−, 4 A−T+ and 92 A+T+ individuals. There were no significant differences in age [*F*(3,322) = 1.02, *P* = 0.40] or years of education [*F*(3,322) = 0.94; *P* = 0.44] across groups. Cognitively impaired (CI) participants exhibited significantly lower Mini-Mental State Examination (MMSE) scores [*t*(324) = 9.12; *P* < 0.001], reduced CSF Aβ42 levels [*t*(240) = 5.43; *P* < 0.001] and elevated Aβ [*t*(324) = 6.01; *P* < 0.001] and tau SUVR [*t*(324) = 5.28; *P* < 0.001] as expected for Alzheimer’s disease-related impairment. Full demographic and biomarker characteristics of the TRIAD cohort are presented in Table 1.

**Table 1:** The complete demographic characteristics of TRIAD cohort utilized in the current study

Characteristic	CU (Y)	A−T−	A + T−	A−T+	A + T+
Number	52	166	64	4	92
Mean age, years (SD)	22.43 (2.01)	67.71 (19.17)	70.45 (15.85)	65 (9.09)	64.09 (8.33)
Female, number (%)	31 (59.61)	104 (62.65)	38 (59.37)	2 (50)	57 (61.95)
Mean education, years (SD)	16.70 (1.72)	15.68 (3.29)	15.28 (3.48)	17 (4.69)	14.84 (3.83)
Mean MMSE score, (SD)	29.75(0.63)	29.18 (2.13)	28.24 (2.41)	20 (1)	22.53 (6.15)
Mean CDR scores, (SD)	0 (0)	0 (0)	0.24 (0.29)	0.87 (0.22)	0.83 (0.56)
APOE ε4, number (%)	10 (19.23)	36 (21.68)	23 (35.93)	1 (25)	53 (57.60)
Total amyloid SUVR^a^, mean (SD)	1.17 (0.10)	1.25 (0.10)	2.04 (0.34)	1.36 (0.11)	2.47 (0.42)
Total tau SUVR^b^, mean (SD)	0.89 (0.10)	0.92 (0.10)	0.99 (0.10)	3.19 (0.74)	2.33 (0.79)

No significant differences in age or years of education were observed across groups. However, CI participants showed significantly lower MMSE scores, decreased CSF Aβ42 levels and increased Aβ and tau SUVR.

^a^Amyloid cutoff = 1.55 SUVR Neocortex.

^b^Tau cutoff = 1.18 SUVR MetaROI.

### Ventricular parameter remodelling in Alzheimer’s disease

**Figure 1:**
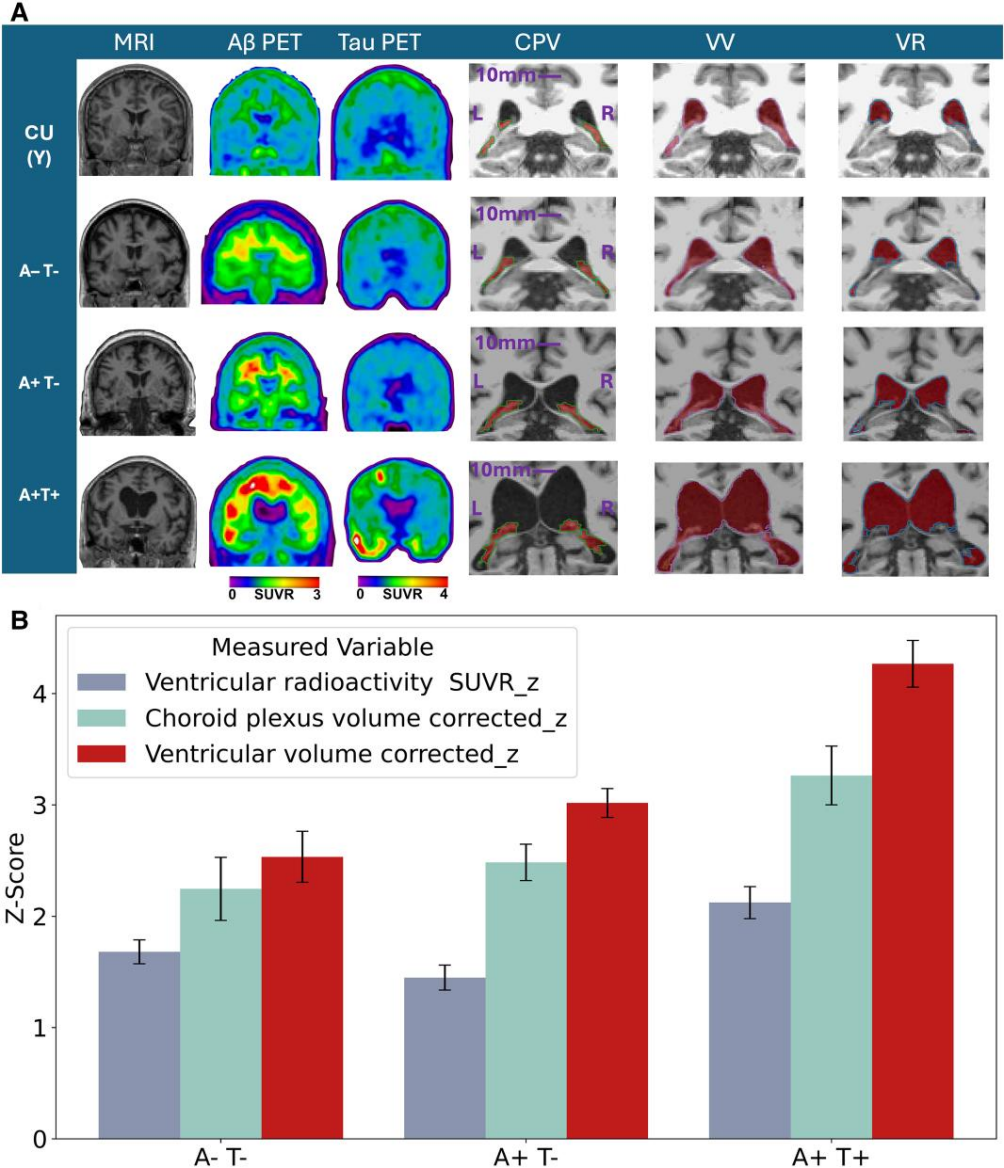
**CSF-related structures’ changes in Alzheimer’s disease**. (**A**) Representative MRI, amyloid-PET ([^18^F]AZD4694), tau-PET ([^18^F]MK6240), CPV, VV and VR across CU(Y), A−T−, A+T− and A+T+ groups. CPV and VV were segmented from MRI, and VR masks were derived from MRI-based VV with CPV excluded and applied to PET images. (**B**) *Z*-score bar plot normalized by the CU(Y) participants under 25 years old and corrected for ICV of ventricular parameters involved in CSF dynamic. The VR SUVR_z was reversed for better visualization. In this plot, error bars represent the standard error of the mean, indicating the variability or precision of the *Z*-score estimate for each bar. Sample sizes were as follows: *n* = 166 A−T−, 64 A+T− and 92 A+T+ individuals.

Ventricular parameter remodelling refers to structural and functional adaptations of the ventricular system, encompassing ventricular enlargement and choroid plexus changes that reflect CSF dynamics and clearance adaptations occurring across ageing and Alzheimer’s disease progression. Figure 1 illustrates group-wise differences in MRI-based CPV and VV segmentations, as well as VR masks applied to amyloid- and tau-PET. Representative images from CU(Y), A−T−, A+T− and A+T+ participants highlight progressive ventricular and choroid plexus remodelling and changes in VR across the Alzheimer’s disease continuum. *Z*-score bar plot of ventricular parameters normalized by the mean and standard deviation of CU(Y) participants and corrected for ICV (before *Z*-score calculation) showed that ventricular remodelling is an ageing-associated event [A−T− older adults (65 years old and over)] and further increases in Alzheimer’s disease (Fig. 1). The strongest change amongst the ventricular remodelling parameters was observed for VV, which showed the highest increase up to *Z*-score 4 in the presence of abnormal loads of both Aβ and tau.

### Choroid plexus function is reduced in Alzheimer’s disease

**Figure 2:**
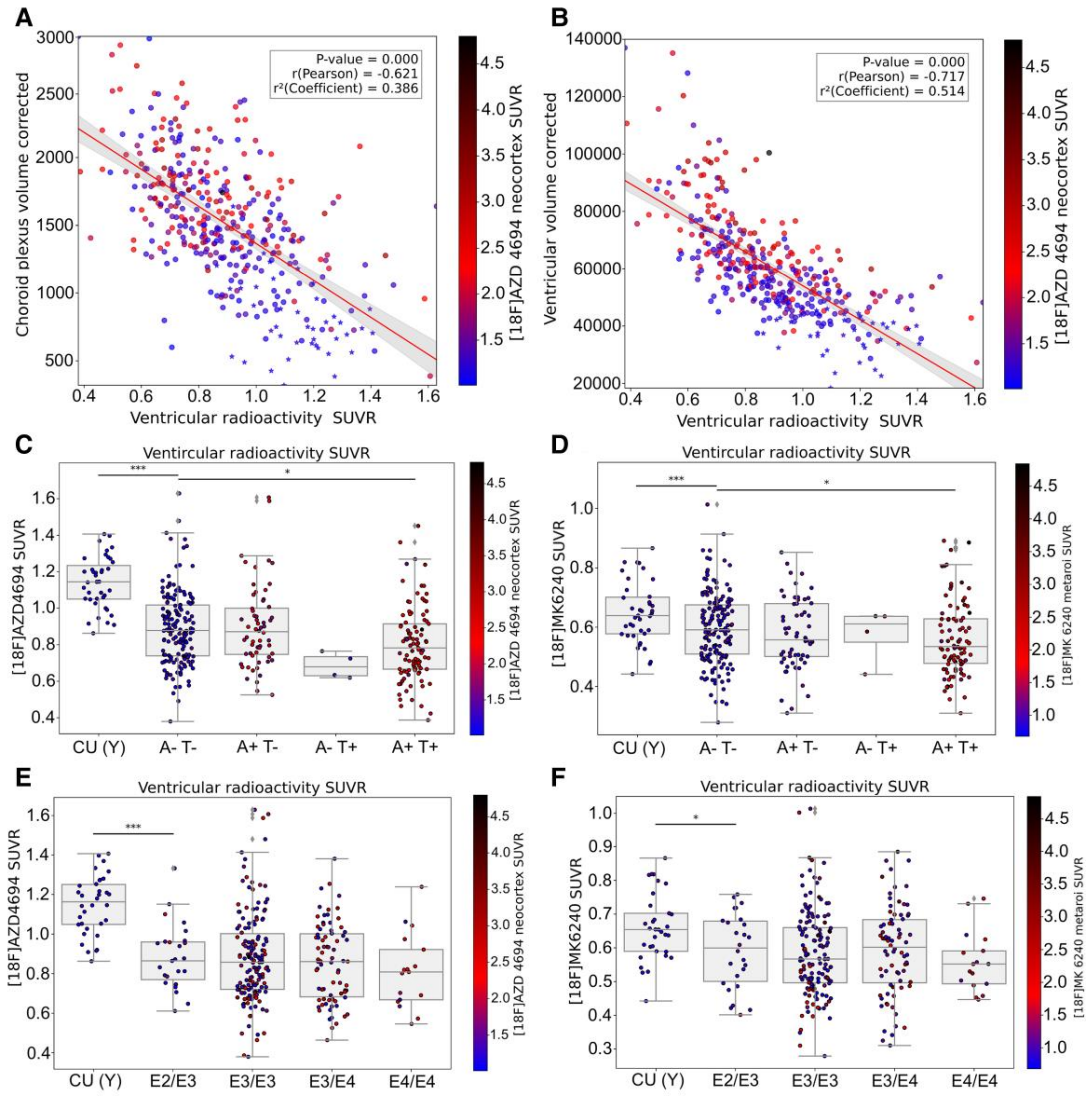
**Choroid plexus function is associated with Alzheimer’s disease pathophysiology**. CPV (*ρ* = −0.62; *P* = 0.001) (**A**) and VV (*ρ* = −0.71, *P* = 0.001) (**B**) (corrected for ICV) were negatively correlated with VR (derived from amyloid-PET). Excluding the CU(Y) from the regression model, CPV (*ρ* = −0.51; *P* = 0.001) and VV (*ρ* = −0.59; *P* = 0.001) were negatively correlated with VR (derived from amyloid-PET). For a better visualization (**A** and **B**), CU(Y) participants were marked with an asterisk (*). Each dot represents one participant (total *N* = 378). Overall, VR decreased in Alzheimer’s disease spectrum, with a significant reduction in the A+T+ group compared to A−T−. These reductions occurred for both PET tracers [amyloid-PET (**C**) and tau-PET (**D**)], similarly. Each dot represents one participant (total *N* = 378). However, the level of VR did not change based on different APOE genotypes (**E** and **F**). APOE genotypes are defined by the ε2 (E2), ε3 (E3) and ε4 (E4) alleles. Each dot represents one participant (*total N* = 356). ‘*’, ‘**’ and ‘***’ represent Tukey *post hoc* tests following ANOVA for pairwise comparisons and FDR (*q* < 0.05)-corrected *P*-values in the following ranges: 0.05–0.01, 0.01–0.001 and <0.001, respectively.

Using VR SUVR derived from [^18^F]AZD4694, we found negative correlation between VR SUVR and CPV and VV (Fig. 2A and B, Spearman’s *ρ* = −0.62 and −0.71, respectively; both *P* < 0.001), meaning that lower VR SUVR was associated with higher CPV and VV. Our results support a direct link between structural characteristics and choroid plexus-related CSF dynamics in Alzheimer’s disease. Also using [^18^F]AZD4694, we observed reduction of VR in CU A−T− compared with CU(Y) (*P* < 0.001) and in A+T+ individuals compared with CU A−T− participants (*P* = 0.02; Fig. 2C). To exclude tracer-specific effects, we then replicated this group-level pattern using the independent PET tracer [^18^F]MK6240 (Fig. 2D). Because [^18^F]AZD4694 and [^18^F]MK6240 differ in pharmacokinetics and are quantified over distinct post-injection time windows, this cross-tracer replication allowed us to assess the robustness of ventricular SUVR findings to tracer- and timing-related effects. Additionally, no significant differences in VR were observed across APOE genotypes (Fig. 2E and F; all *P* > 0.05), and this null effect was consistent for both [^18^F]AZD4694 and [^18^F]MK6240.

### Ventricular volume and choroid plexus function are associated with Alzheimer’s disease pathology

**Figure 3:**
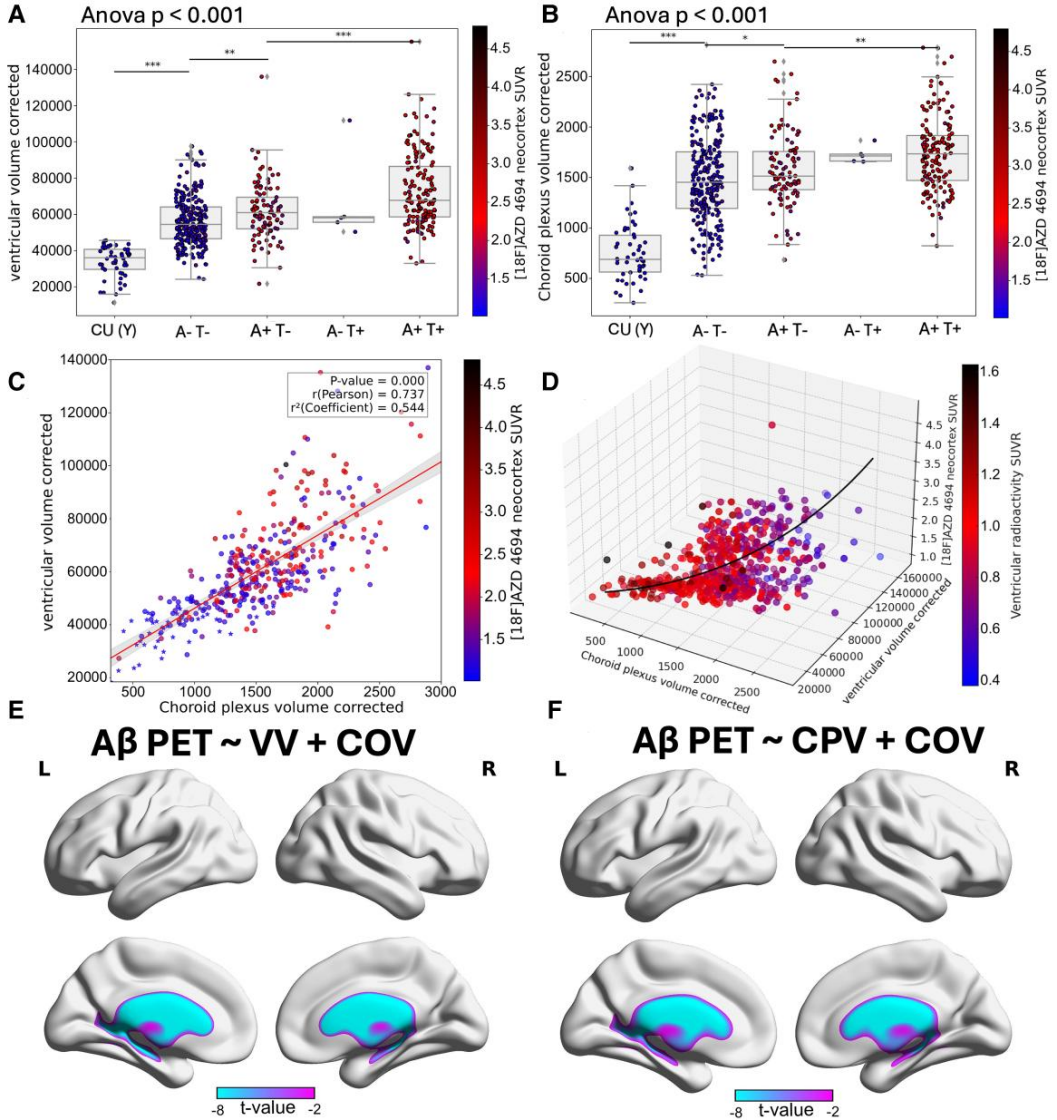
**Choroid plexus and ventricular remodelling in Alzheimer’s disease spectrum**. VV (**A**) and CPV (**B**), corrected for ICV based on different amyloid and tau statuses and colour coded based on the [^18^F]AZD4694 SUVR in the neocortex. The relationship between CPV and VV was colour coded based on the [^18^F]AZD4694 SUVR in the neocortex (**C**), including CU(Y) participants (*ρ* = 0.74; *P* = 0.001). When CU(Y) participants were excluded from the regression model, CPV and VV remained correlated (*ρ* = 0.63; *P* = 0.001). For a better visualization in **C**, CU(Y) participants were marked with an asterisk (*). Each dot represents one participant (*total N* = 378). 3D visualization to include all three ventricular parameters that contribute to amyloid aggregation, in the model (covariates = age, sex, APOE4, ICV): Neocortical Aβ ∼ CPV + VV, colour coded based on the concentration level of radioactivity in the ventricle (derived from amyloid-PET) (**D**). Voxel-wise analysis results for the older adult groups (65 years old and over), visualized as T-statistical parametric maps (FDR corrected for multiple comparisons at *P* < 0.001) overlaid on a structural template indicating regions where linear regression revealed a significant negative association between amyloid-PET and VV and CPV in the ventricles (**E** and **F**). Covariates (COV) = age, sex, APOE4, ICV. ‘*’, ‘**’ and ‘***’ represent Tukey *post hoc* tests following ANOVA for pairwise comparisons and FDR (*q* < 0.05)-corrected *P*-values in the following ranges: 0.05–0.01, 0.01–0.001 and <0.001, respectively.

We found a significant main effect of group on VV [ANOVA, *F*(4,321) = 18.42, *P* < 0.001; Fig. 3A]. *Post hoc* Tukey tests revealed that VV was significantly greater in A−T− compared to CU(Y) participants (*P* < 0.001) and further increased in A+T− (*P* < 0.001) and A+T+ groups (*P* < 0.001). Similarly, there was a significant main effect of group on CPV [ANOVA, *F*(4,321) = 11.87, *P* < 0.001; Fig. 3B]. CPV was significantly higher in A−T− compared with CU(Y) (*P* < 0.001) and showed progressive enlargement in A+T− (*P* = 0.008) and A+T+ (*P* < 0.001). These two ventricular parameters were highly correlated with each other as shown in Fig. 3C. After correcting for ICV, CPV and VV had a Spearman correlation (*ρ*) of 0.74 and a *R*^2^ equal to 0.54.

### Ventricular volume is associated with amyloid-beta cortical aggregation

We next aimed to test the combined effects of both ventricular parameters to predict the abnormal aggregation of Aβ in the neocortex by performing a linear model corrected for covariates, visualized in a three-dimensional representation (Fig. 3D). In the model [covariates (COV) = age, sex, APOE4, ICV]:


NeocorticalAβ∼choroidplexusvolume+Ventricularvolume+COV.

We observed a coefficient of determination *R*^2^ = 0.45. Within the model, the coefficient of CPV was equal to 0.04 [*β*_STD_ = 0.04, 95% CI (0.00, 0.14), *P* < 0.001], showing for every one unit increase in CPV, the global amyloid aggregation in the cortex is expected to increase by 0.04 SUVR, holding the VV constant. The coefficient of VV was equal to 0.01 [*β*_STD_ = 0.01, 95% CI (0.00, 0.11), *P* < 0.001], meaning for every one unit increase in the VV, the global amyloid aggregation is expected to increase by 0.01 SUVR, holding the CPV constant. Therefore, both volumes were significantly and independently predictive of amyloid-PET neocortical signal.

To test the effect of VV and CPV on VR, we conducted a voxel-wise analysis between amyloid-PET and VV and CPV separately. This analysis was conducted in a subsample including older individuals only (>65 years old) in order to remove the ageing bias. In both cases, we observed significant negative associations inside the ventricle (Fig. 3E and F).

**Figure 4:**
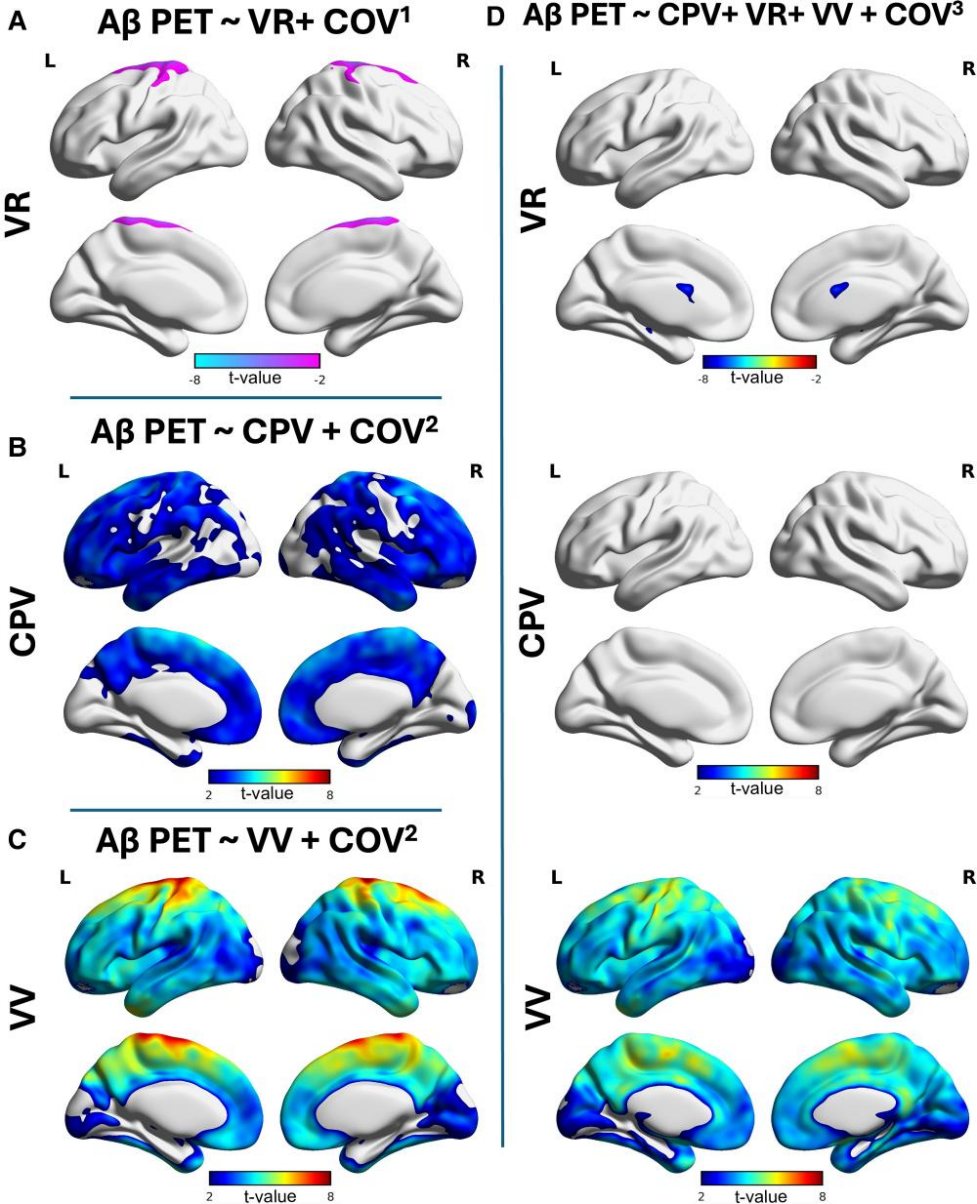
**Ventricular enlargement is the main contributor to amyloid aggregation amongst the ventricular parameters**. Voxel-wise analysis results for the older adult groups (65 years old and over, *N* = 265), visualized as T-statistical parametric maps (FDR corrected for multiple comparisons at *P* < 0.001) overlaid on a structural template indicate regions where linear regression revealed a significant negative association between amyloid-PET and VR SUVR (VR) (derived from amyloid-PET) (**A**), and significant positive association between amyloid-PET and CPV and VV (**B** and **C** respectively). After correcting the effect of these parameters on Aβ, VV carries all the effect on amyloid aggregation (**D**). COV^1^ = age, sex, APOE4. COV^2^ = age, sex, APOE4, ICV. COV^3^ = age, sex, APOE4, ICV, grey matter volume.

When looking at voxel-wise amyloid-PET in older adult groups (*N* = 265, 65 years old and over) we found that, when considered independently, all three ventricular parameters were significantly associated with the cortical amyloid-PET signal. Firstly, VR showed strong negative associations in the dorsal apex of the neocortex (Fig. 4A). Secondly, CPV showed moderate positive associations throughout the cortex (Fig. 4B). Thirdly, VV showed significant positive associations across the cortex (Fig. 4C). However, when including all three ventricular parameters in the same model, only the VV remained positively and significantly associated with amyloid-PET across the neocortex, while the associations with CPV and VR were no longer significant (Fig. 4D). The VV may therefore be the most important ventricular parameter predicting amyloid-PET throughout the neocortex.

### Neocortical amyloid-beta mediates the effects of ventricular volume on tau cortical aggregation

**Figure 5:**
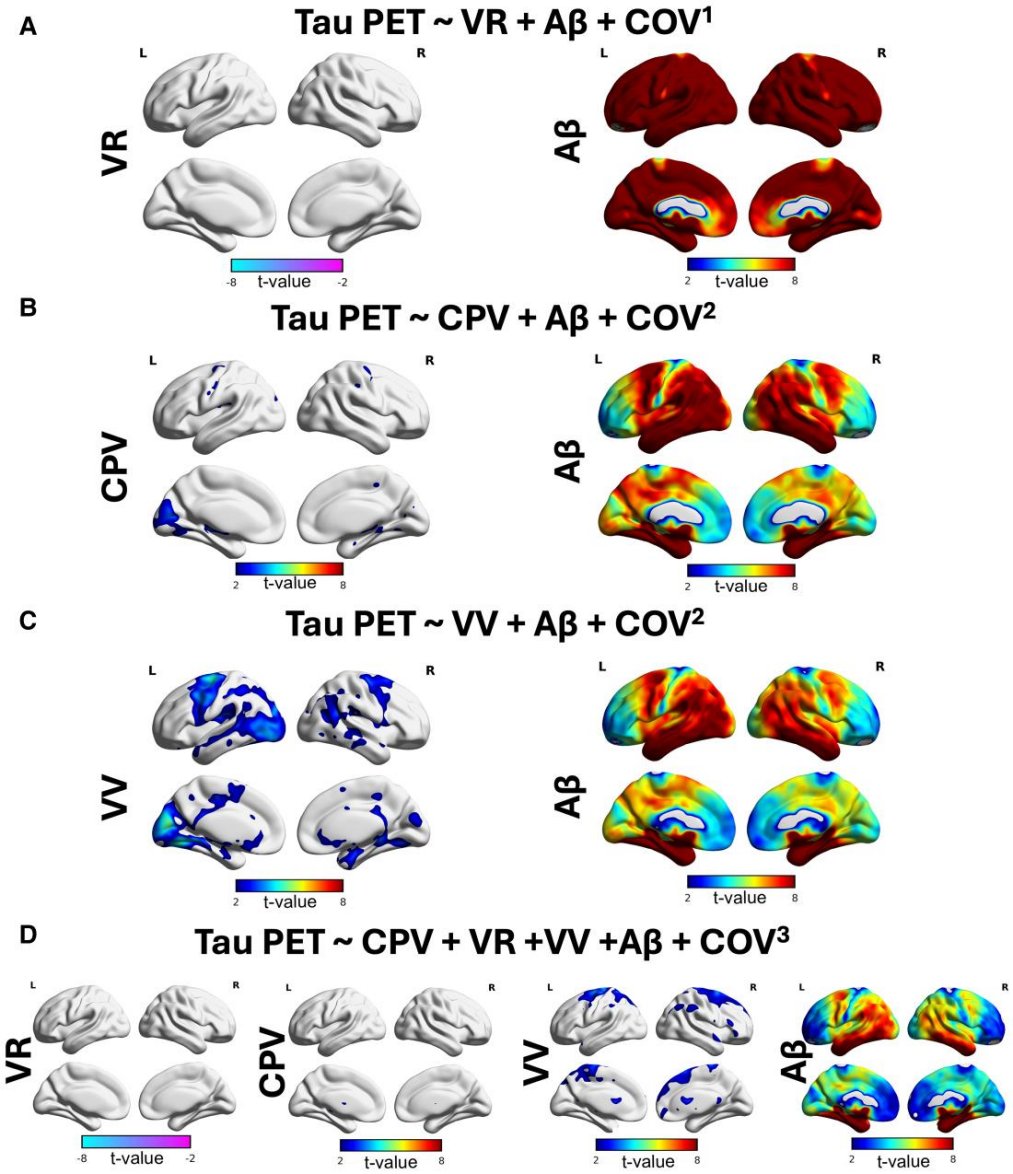
**Amyloid mediates the effect of ventricular enlargement on tau load**. Voxel-wise analysis results for the older adult groups (65 years old and over, *N* = 265), visualized as T-statistical parametric maps (FDR corrected for multiple comparisons at *P* < 0.001) overlaid on a structural template indicate regions, where linear regression revealed Aβ carries the effect of VR (derived from tau-PET) (**A**) and CPV (**B**) on tau load. However, linear regression showed a significant positive association between VV and tau load after correcting for amyloid (**C**), which disappears after correcting for other ventricular parameters and the effect solely carries by Aβ (**D**). COV^1^ = age, sex, APOE4. COV^2^ = age, sex, APOE4, ICV. COV^3^ = age, sex, APOE4, ICV, grey matter volume.

We next aimed to predict the tau load distribution using individual ventricular parameters while considering the global neocortical Aβ in models. We first found that VR (Fig. 5A) and CPV (Fig. 5B) were not significantly associated with tau-PET, and their effect was completely carried by neocortical Aβ. However, VV showed significant positive associations in the posterior cingulate, precuneus, lateral temporal cortex, superior frontal gyrus and occipital cortex despite the strong neocortical Aβ associations with tau-PET all over the cortex. When including the three ventricular parameters in the same model, neocortical Aβ carried the entire effect of tau-PET, further showing that the effect of ventricular parameters might be mediated by amyloid-PET.

**Figure 6:**
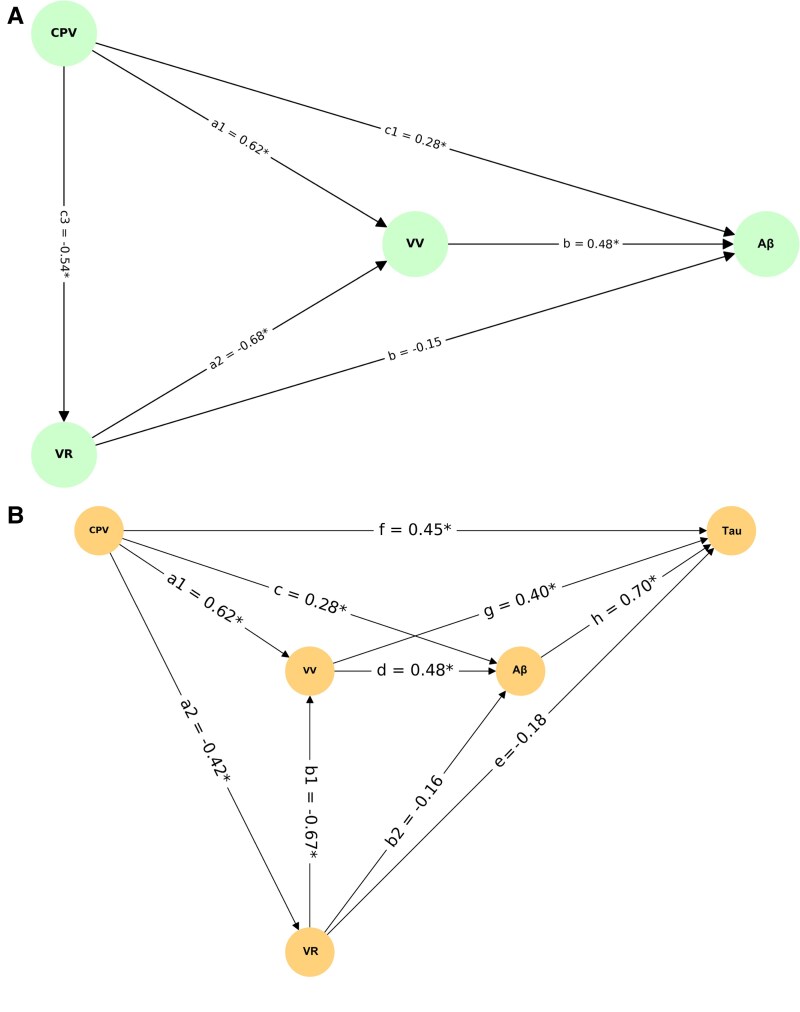
**VV is the mediator of ventricular parameters on Aβ, and Aβ is the mediator of all of them on tau load**. Pathway mediation analysis shows VV is a significant mediator of the direct effect of ventricular parameters on Aβ (**A**). Path a1 denotes the effect of CPV on VV; a2 denotes the effect of VR on VV; b denotes the effect of VV on neocortical Aβ; the additional b path denotes the direct effect of VR on Aβ; c1 denotes the direct effect of CPV on Aβ; and c3 denotes the direct effect of CPV on VR. Regarding their effect on tau, Aβ mediates the effect of VV on tau load (**B**). Path a1 denotes the effect of CPV on VV; a2 denotes the effect of CPV on VR; b1 denotes the effect of VR on VV; b2 denotes the direct effect of VR on neocortical Aβ; c denotes the direct effect of CPV on Aβ; d denotes the effect of VV on Aβ; f denotes the direct effect of CPV on tau; g denotes the effect of neocortical VV on tau; and h denotes the direct effect of Aβ on tau. All models were adjusted for age, sex, APOE ε4 carriership and ICV. Asterisks (*) indicate statistically significant paths (*P* < 0.05).

We then aimed to verify if VV mediates the effect of the other two ventricular parameters on amyloid-PET SUVR, which, in turn, would mediate the effect of ventricular parameters on tau-PET SUVR. To test this, we performed two-path analyses. The first path quantifies the extent to which VV mediates the association between CPV or VR and neocortical amyloid-PET SUVR, whereas the second path examines whether neocortical amyloid-PET in turn mediates the link between these ventricular parameters and temporal tau-PET SUVR. Indirect effects were estimated using bias-corrected bootstrapping (10 000 iterations), with significance defined by 95% confidence intervals not crossing zero. This analytical approach captures sequential biological dependencies. In the first one, we found that the VV significantly mediated the relationship of both the VR and the CPV on the neocortical amyloid-PET signal (Fig. 6A). In the second one, we added temporal tau-PET signal as the predicted variable. Here, we found that neocortical amyloid-PET significantly mediated the relationship between ventricular parameters and temporal tau-PET (Fig. 6B).

## Discussion

### Summary of the results

CSF dynamics encompass the production of CSF in the choroid plexus, the circulation within the ventricles and the subarachnoid space and finally the reabsorption of CSF, which plays a critical role in brain homeostasis, amongst others by ensuring elimination of brain metabolic waste.^45^ While the choroid plexus remains the main site of CSF production, a smaller portion is generated by cerebral capillaries and through bidirectional water exchange across the blood–brain barrier, all of which collectively contribute to CSF homeostasis and clearance dynamics.^46^

It has been proposed that perturbations in this clearance mechanism facilitate the development of brain amyloidosis.^47^ In the present study, we focused on the combined analysis of the CPV and VV as determinants of cortical Aβ and tau load. By integrating the CPV and VV with PET imaging markers of Aβ and tau, we provided a perspective on how alterations in CSF dynamics are associated with early Alzheimer’s disease pathology. First, we found that all the ventricular parameters were independently associated with amyloid-PET signal, individually. Second, we found that VV had the largest effect on Aβ load, and that it had a mediation effect on CPV and function as measured by VR. Third, we found that ventricular parameters were not directly associated with tau load, and their effects were mediated by Aβ.

### Choroid plexus function

To indirectly assess CSF dynamics, we measured PET signal in the lateral ventricles, reflecting radiotracer diffusion within the CSF compartment. Although this measure cannot directly isolate choroid plexus secretory capacity, it provides a proxy for CSF turnover and clearance efficiency.^48^ PET signal within the CSF compartment has previously been measured and used to investigate CSF flow and clearance dynamics using dynamic PET approaches.^12,13^ VR mainly reflects the passive diffusion of unbound tracer molecules within the CSF. In individuals with larger ventricles, under a steady-state condition, this signal indicates altered CSF flow dynamics or reduced turnover efficiency.^12,13^ We excluded choroid plexus from the ventricle for VR calculation to control for any kind of possible off-target binding of different PET tracers.^49^ Previous methodological studies using dynamic PET have shown that ventricular signal fluctuations correspond to CSF turnover and clearance efficiency rather than specific tracer binding.^12,13^ These studies support static PET-derived ventricular SUVR as a surrogate for CSF dynamics, albeit with limited temporal resolution. To examine our hypothesis, we calculated the differences of VR across A/T stages and observed significant reductions in the presence of both amyloid and tau in the brain. Our results are in line with previous finding showing VR,^12-14^ and choroid plexus function^11^ are declining in Alzheimer’s disease.

### Choroid plexus enlargement

Umemura *et al*.^50^ demonstrated that choroid plexus enlargement in an elderly population is an independent predictor of MCI and might be a specific biomarker for early Alzheimer’s disease diagnosis. Their results underscored that enlargement of the choroid plexus performs better than cortical atrophy in predicting cognitive decline. Our results are aligned with these findings, since they suggest that choroid plexus enlargement is linked to ageing and furthers increases in the presence of Aβ and tau. Jiang *et al*.^51^ extended these results showing that choroid plexus enlargement is associated with Alzheimer’s disease progression, serving as a novel neuroimaging marker for early detection and prognosis. They illustrated that the choroid plexus enlargement is correlated with decreased cognitive scores, effectively differentiating between MCI, Alzheimer’s disease dementia and healthy controls; therefore, it might enhance diagnostic accuracy by supplementing traditional imaging biomarkers, such as hippocampal measures.

### Ventricular remodelling

Our results revealed that ventricular enlargement is associated with protein aggregation in the brain. We suspect that these associations may be due to the CSF dynamics: the production, circulation and its optimum functionality in the elimination of metabolic waste products, such as Aβ proteins.^52^ Our results are consistent with numerous previous findings. For example, Nestor *et al*.^15^ revealed that ventricular enlargement is a sensitive marker for detecting neuropathological changes in MCI and Alzheimer’s disease, providing a feasible metric for assessing disease progression in multi-centre studies. They highlighted significant ventricular changes over 6 months in subjects with MCI and AD and the potential for reduced sample sizes in studies using ventricular measurements compared to cognitive scores. Enlargement of the ventricles is believed to exert hydrostatic pressure on the choroid plexus and other CSF-producing structures, thereby potentially stimulating compensatory CSF secretion.^23,53^ However, the function of the choroid plexus may also be compromised in AD. This dual scenario, ventricular enlargement and compromised choroid plexus function (which may also manifest as choroid plexus hypertrophy^54^), coexist in the same patient, potentially creating a complex interplay amongst CSF-related abnormalities. These abnormalities may disrupt normal CSF dynamics, leading to downregulation of CSF’s physiological reabsorption processes leading to an environment that fosters rapid deposition of Aβ within the cortical parenchyma and perivascular spaces.^55^

Li *et al*.,^14^ using an approach similar to ours, investigated the role of CSF clearance in Alzheimer’s disease using PET tracers. Although they did not consider the mediation effect of choroid plexus and VV enlargement on Aβ aggregations, they showed that impaired CSF clearance is significantly associated with increased brain Aβ deposition, reduced cortical thickness and decreased cognitive performance in Alzheimer’s disease patients. Their results are in line with the framework in which CSF dynamic abnormality contributes to Alzheimer’s disease pathology. Simon and Iliff^56^ described the impact of CSF abnormal dynamics in metabolite accumulation and overall central nervous system dysfunction. They showed that the dysfunction of the glymphatic system, which facilitates CSF–interstitial fluid exchange, has been strongly linked to impaired clearance of Aβ and other neurotoxic metabolites, contributing to neurodegeneration and Alzheimer’s disease progression.

### Ventricular enlargement is the major cerebrospinal fluid-related abnormality associated with protein aggregation

Using multimodal approaches, Todd *et al*.^17^ explored the relationship between both ventricular enlargement and periventricular changes with cognitive decline in ageing, using longitudinal MRI. Their findings also indicated that ventricular enlargement and periventricular oedema correlate with cognitive impairment severity, suggesting that impaired clearance mechanisms contribute to pathophysiological changes in ageing and CI individuals. However, a recent human and mouse study focused on proteomic profiles identified independent age-related and Alzheimer’s disease-related dysregulated protein pathways involved in cell function and architecture in the choroid plexus.^57^ Our results help explain these findings, as we found that the effects of morphofunctional choroid plexus alterations are mediated by ventricular enlargement on Aβ aggregation, which was not considered in their study.

The negative association between CSF radioactivity and cortical Aβ load supports the notion that choroid plexus CSF synthesis capacity is compromised in Alzheimer’s disease. The choroid plexus plays a crucial role in producing and maintaining the composition of CSF and volume within the central nervous system. By contrast, although affecting CSF volume and flow, ventricular enlargement has negligible impact on CSF composition.

The apparent effects of ventricular or choroid plexus changes on tau pathology are mediated by Aβ rather than by direct association. In this case, the lack of a strong relationship between the progress of ventricular remodelling and development of tau pathology might be attributed to the nature of tau deposition across brain regions. Aβ pathology contributes to tau pathology by initiating inflammation and/or neuronal damage.^58^ Therefore, even though ventricular remodelling and Aβ deposition co-occur, the effects of ventricular remodelling on tau are secondary to the processes initiated by Aβ.^59^ The results obtained in the present study support the involvement of the choroid plexus and ventricular system in the formation of Aβ plaques. The choroid plexus might be a key figure not only in terms of CSF processes but also as a regulator of the biochemical environment of the brain. Our data highlight that amyloid and tau accumulation arise from distinct mechanisms, suggesting that enhancing ventricular clearance could be particularly effective in facilitating amyloid removal. The observed relationships between CPV, ventricular enlargement and Aβ deposition further justify exploring therapeutic strategies aimed at improving choroid plexus function.

In the current study, EI was applied as a conservative imaging-based criterion to exclude participants with disproportionate ventriculomegaly that could reflect secondary hydrocephalus rather than Alzheimer’s-related remodelling. A threshold of EI > 0.3 was not intended as a diagnostic marker but as a safeguard against including subjects with mechanical ventricular dilation unrelated to disease pathology. Notably, all excluded participants (*n* = 12, ≈2%) were A− T− CU, consistent with non-Alzheimer’s disease ventriculomegaly patterns. A key strength of our approach lies in the adjustment for neurodegeneration in our measurements while accounting for all relevant confounding factors, including age, sex and APOE4 status. First, we corrected VV by ICV, ensuring that variations in ICV did not confound our analyses. Secondly, when performing voxel-wise analyses, we used grey matter volume as covariate to differentiate effects of age-associated ventricular enlargement from Alzheimer’s disease-related neurodegeneration. This dual adjustment strategy enables us to dissect the independent and specific effects of VV on Alzheimer’s disease pathology, enhancing the precision and validity of our findings. Because participants were grouped according to A/T biomarker stages rather than clinical diagnosis, and in line with the updated NIA-AA research framework,^60^ individuals with A+ are already considered within the Alzheimer’s disease spectrum.

The present study expands the interpretation of ventricular enlargement by demonstrating association between VV and cortical Aβ burden. This relationship suggests that ventricular enlargement may reflect impaired CSF dynamics, potentially hindering the clearance of Aβ from the interstitial and perivascular spaces. In this context, the observed reduction in VR (interpreted as a proxy for diminished choroid plexus function and reduced CSF turnover) further supports the notion that inefficient CSF renewal and clearance pathways contribute to amyloid accumulation in the cortex. Together, these findings corroborate the notion that both structural (ventricular enlargement) and functional (choroid plexus dysfunction) alterations of the choroidal–ventricular system may contribute to Aβ deposition through impaired CSF-mediated clearance mechanisms.

Our results should be supplemented by future studies in several ways. First, although VR serves as a proxy for CSF flow, it does not capture real-time clearance kinetics. More advanced imaging techniques, such as dynamic PET or MRI-based CSF flow mapping, could complement our approach. Second, as a single-centre study, the impact of multi-centre and multi-scanner should be evaluated. Third, the demographics of the TRIAD cohort are not representative of the population diversity in North America or all over the world. Therefore, studies with more representative cohorts are needed to further investigate. Fourth, patients with multiple comorbidities, particularly with neurological conditions, were systematically excluded from this study, and as many neurodegenerative conditions also involve abnormal deposition of protein aggregates, their impact on the parameters measured here needs to be assessed. Fifth, although EI provides a simple linear estimate of ventricular size, it lacks volumetric precision and clinical specificity. Notably, EI values exceeding 0.3 have been reported in approximately one-fifth of apparently cognitively normal older adults,^26^ underscoring the limited specificity of this threshold in ageing populations. However, sensitivity analyses in our study demonstrated that exclusion based on this criterion did not alter the direction or significance of the observed associations, indicating that our findings were not driven by this methodological decision. Future studies incorporating complementary imaging features (e.g. callosal angle, full ventricular volumetry, or CSF flow metrics) and clinical assessments will further refine approaches to characterizing abnormal ventricular enlargement and CSF-related alterations.^26,61-65^ Finally, FreeSurfer segmentations were not subjected to systematic manual quality control across all participants, which may have introduced additional variability and unreliability, particularly in the choroid plexus due to limited boundary precision compared to specialized segmentation methods (e.g. ASCHOPLEX^66^ or deep learning–based^67^ approaches).

## Conclusion

Our results support the notion that choroid plexus and ventricular remodelling are associated with early Alzheimer’s disease-related changes, beginning during the A− T− to A+T− transition and becoming more pronounced with both Aβ and tau pathology. CSF production, regulation and turnover might underlie this relationship, leading to reduced brain clearance of toxic protein deposits. This study might support novel pharmacological and non-pharmacological therapeutic approaches focused on improving the regulation of CSF dynamics.

## Supplementary Material

fcag066_Supplementary_Data

## Data Availability

All requests for raw and analysed data and materials will be promptly reviewed by McGill university to verify if the request is subject to any intellectual property or confidentiality obligations. Anonymized data will be shared upon request to the study’s senior author from a qualified academic investigator for the sole purpose of replicating the procedures and results presented in this article. Any data and materials that can be shared will be released via a material transfer agreement. Data are not publicly available due to information that could compromise the privacy of research participants. Related documents, including study protocol and informed consent forms, can similarly be made available upon request. All scripts used for data preprocessing, statistical analyses and figure generation are openly available in our online code repository: https://github.com/TNL-MCSA/Tutorials/wiki.
