# Supplementary material for: Ventricular enlargement is associated with early Alzheimer’s disease pathophysiology
Source: Brain Commun. 2026 Mar 7;8(2):fcag066. doi: 10.1093/braincomms/fcag066 (PMC12993450; doi:10.1093/braincomms/fcag066)
Supplement: fcag066_Supplementary_Data [file fcag066_supplementary_data.docx]

**Ventricular enlargement is associated with early Alzheimer’s disease pathophysiology**

Seyyed Ali Hosseini, Etienne Aumont, Nesrine Rahmouni, Marcel S. Woo, Arthur C. Macedo, Brandon Hall, Lydia Trudel, Tevy Chan, Jaime Fernandez Arias, Yi-Ting Wang, Stijn Servaes, Joseph Therriault, Yansheng Zheng, Kely Quispialaya Socualaya, Gleb Bezgin, Cécile Tissot, Delphine Oliva-Lopez, Robert Hopewell, Chris Hung-Hsin Hsiao, Catherine Saleh, Jenna Stevenson, Firoza Lussier, Liyong Wu, Min Chu, Sanjeev Chawla, Vladimir Fonov, Gassan Massarweh, Yasser Iturria-Medina, Jean-Paul Soucy, David A. Rudko, Serge Gauthier, Thomas Karikari, Andréa Lessa Benedet, Nicholas J. Ashton, Henrik Zetterberg, Maxime Montembeault, Paolo Vitali, Kaj Blennow, D. Louis Collins, Jesse Klostranec, Tharick A. Pascoal, Pedro Rosa-Neto*

**Evans’ Index measurement**

Evans’ Index (EI) was calculated from the FreeSurfer segmentation outputs using a semi-automated Python workflow. The lateral ventricles were identified from the aparc+aseg.mgz file (labels 4 and 43 for the left and right lateral ventricles, respectively). For each participant, the algorithm identified the axial slice containing the maximal frontal horn width within the anterior third of the ventricular mask. On that slice, the maximum left-right distance between the combined width of the right and left frontal horns was computed in millimetres using the voxel dimensions in the FreeSurfer header.

To obtain the internal skull diameter (denominator of the EI), the corresponding axial slice from the head-included T1 MRI volume was thresholded and morphologically processed to delineate the inner table of the calvarium. The largest contiguous extent along the left-right axis represented the inner skull width.

Evans’ Index was then computed as:

$$EI= \frac{Frontal horn width (mm)}{Internal skull diameter (mm)}$$

All measurements were visually inspected on the extracted slice to ensure correct delineation of the frontal horns and inner skull boundary.


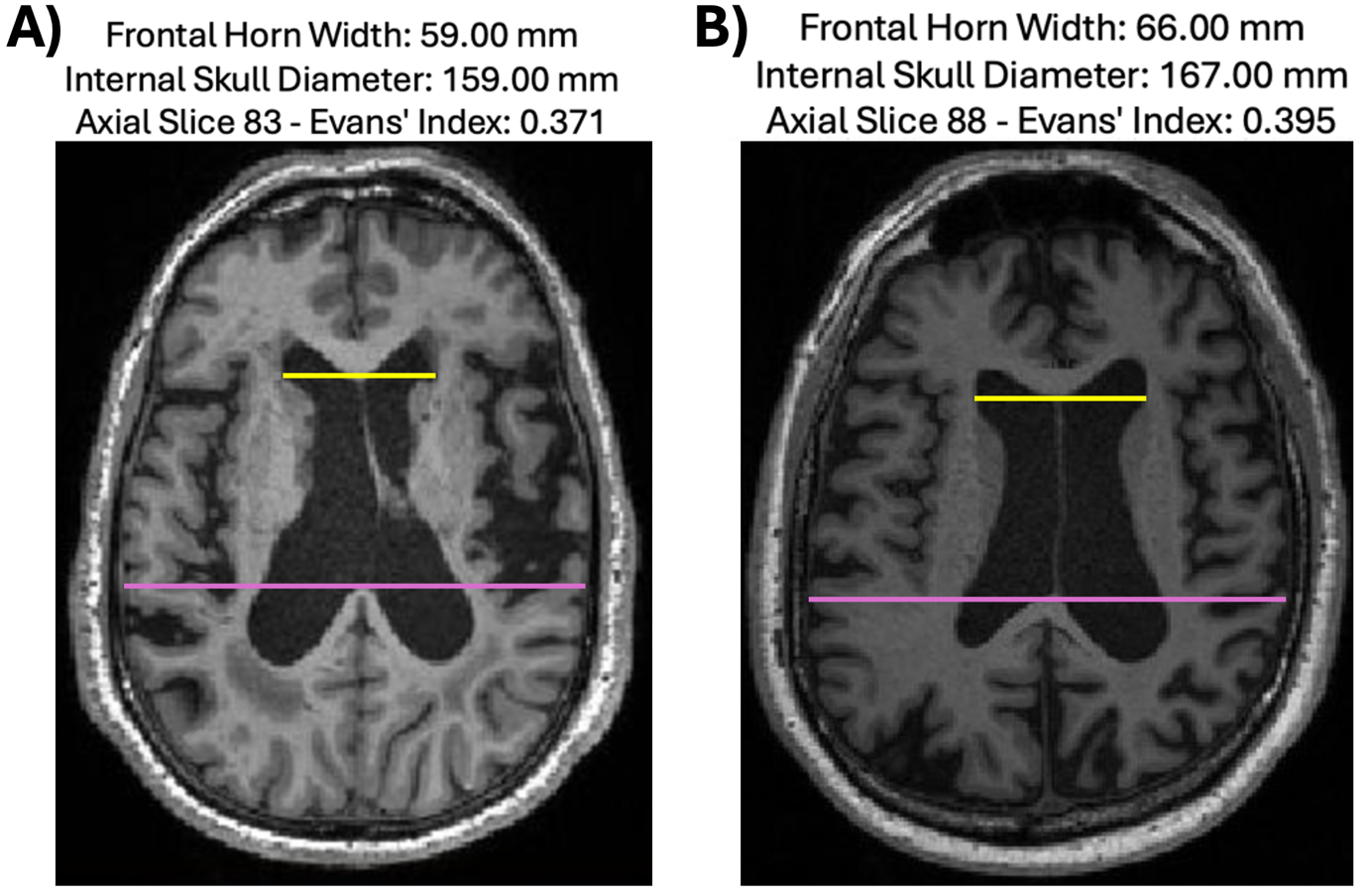


Supplementary Figure 1. **Examples of subjects with Evans’ Index (EI) greater than 0.3.**

Representative axial T1-weighted MR images from two cognitively normal, amyloid-negative tau negative (A-T-) participants with abnormal ventricular enlargement (Evans’ Index > 0.3). For each subject, measurements were performed on a single axial slice at the level of the frontal horns. The frontal horn width (numerator of the Evans’ Index) is depicted by the upper horizontal line, measured as the maximal distance between the lateral borders of the frontal horns of the lateral ventricles. The internal skull diameter (denominator of the Evans’ Index) is depicted by the lower horizontal line, measured as the maximal intracranial width between the left and right inner boundaries of the skull on the same axial slice. Panel A shows a subject with a frontal horn width of 59.0 mm, an internal skull diameter of 159.0 mm, and an Evans’ Index of 0.371 (axial slice 83). Panel B shows a subject with a frontal horn width of 66.0 mm, an internal skull diameter of 167.0 mm, and an Evans’ Index of 0.395 (axial slice 88).


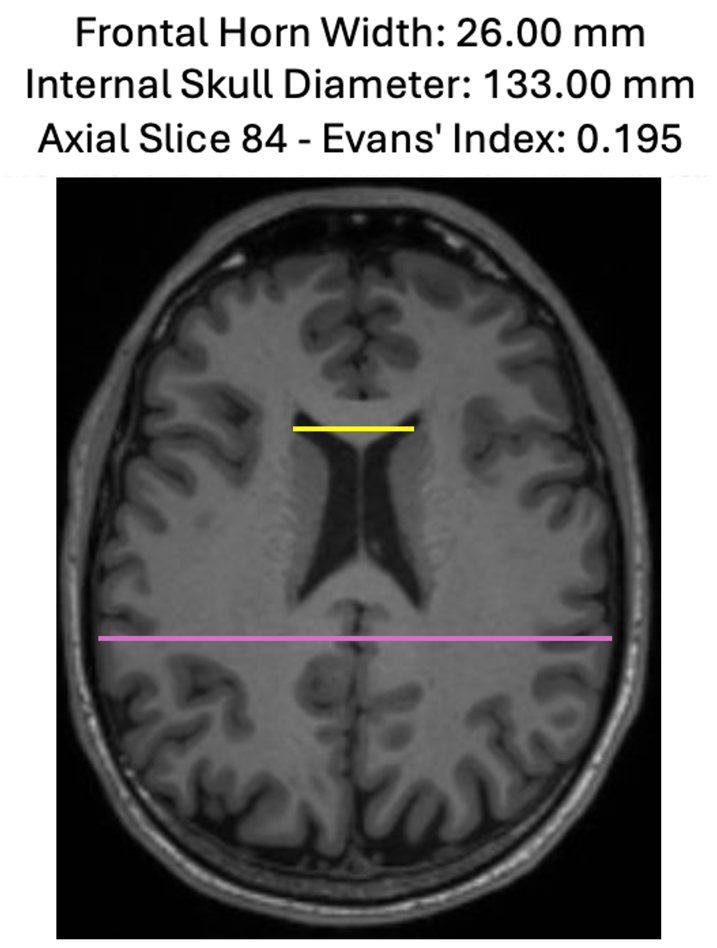


Supplementary Figure 2. **Example of a subject with Evans’ Index (EI) less than 0.3.**

Representative axial T1-weighted MRI from a cognitively normal, amyloid-negative tau negative (A-T-) participant with a normal ventricular size (Evans’ Index < 0.3). Measurements were performed on a single axial slice at the level of the frontal horns. The frontal horn width (numerator of the Evans’ Index) is shown by the upper horizontal line, measured as the maximal distance between the lateral borders of the frontal horns of the lateral ventricles. The internal skull diameter (denominator of the Evans’ Index) is shown by the lower horizontal line, measured as the maximal intracranial width between the left and right inner boundaries of the skull on the same axial slice. For this subject, the frontal horn width measured 26.0 mm, the internal skull diameter measured 133.0 mm, yielding an Evans’ Index of 0.195 (axial slice 84). This value falls within the normal range, illustrating a typical ventricular size in a cognitively normal individual.
